# Awareness of Human Papillomavirus among Male and Female University Students in Saudi Arabia

**DOI:** 10.3390/healthcare11050649

**Published:** 2023-02-23

**Authors:** Esraa Aldawood, Lama Alzamil, Layla Faqih, Deemah Dabbagh, Sarah Alharbi, Taghreed A. Hafiz, Hassan H. Alshurafa, Wajd F. Altukhais, Rufaidah Dabbagh

**Affiliations:** 1 Department of Clinical Laboratories Sciences, College of Applied Medical Sciences, King Saud University, Riyadh 12372, Saudi Arabia; 2 College of Medicine, King Saud University, Riyadh 11461, Saudi Arabia; 3 Department of Family and Community Medicine, College of Medicine, King Saud University, Riyadh 11461, Saudi Arabia

**Keywords:** HPV, awareness, knowledge, health colleges

## Abstract

Background: Human papillomavirus (HPV) is a common sexually transmitted infection globally. Investigating HPV awareness can reduce the burden of HPV-related cancers. Aims: (1) Assessing HPV awareness and knowledge among health college students at King Saud University, (2) comparing these outcomes across sociodemographic characteristics. Methods: A cross-sectional survey study was conducted from November to December 2022 and included 403 health college students. Associations of HPV awareness and knowledge with sociodemographic characteristics were assessed using logistic regression analysis and linear regression analysis, respectively. Results: Only 60% of students were aware of HPV, with awareness higher among females, although their knowledge scores were comparable to males. The odds of awareness of HPV were greater among medical students compared to other colleges and among students belonging to older age groups compared to the younger age group (18–20). The odds of HPV awareness among hepatitis B vaccinated students were 2.10 times that among unvaccinated students (AOR = 2.10; 95% CI = 1.21, 3.64). Conclusions: The low level of HPV awareness among college students warrants the need for HPV educational campaigns to improve HPV awareness and to promote HPV vaccination in the community.

## 1. Introduction

Human papillomavirus (HPV) infection is the most prevalent sexually transmitted infection (STI) among sexually active men and women worldwide. HPV infects the squamous cell lining the inner surface of the cervix, oropharynx, anus, penis, vagina, and vulva [1]. It is responsible for a variety of cutaneous and mucosal epithelial lesions, as well as 15 different cancers, including cervical, penile, anal, and oropharyngeal cancers [1,2,3].

Cervical cancer is considered the fourth most common cancer among females [1]. A pap smear is usually the gold standard for cervical cancer screening worldwide; however, due to sociocultural factors, the screening test coverage in Saudi Arabia is lower than in most countries [4]. Although data on the prevalence of HPV infection and subsequently associated cancers are limited in Saudi Arabia, reported cancer statistics for the year 2020 showed an estimated number of 358 annual new cervical cancer cases in Saudi Arabia, with a crude mortality rate of 1.22 per 100,000 women per year [5].

In recent years, the number of cervical cancer cases in developed countries has significantly dropped due to increased awareness of HPV infection and its vaccination [6]. Studies found that HPV awareness and knowledge are important predictors for HPV vaccine acceptability [7,8]. Moreover, it has been reported that physicians can positively influence parents’ decisions regarding their children’s HPV vaccine uptake [9]. Therefore, educating future healthcare providers about HPV infection is crucial for HPV prevention.

Globally, awareness of HPV is higher among healthcare professionals compared to the general population [10,11]. In addition, studies indicate that factors such as increasing age, being female, and higher education level are positively correlated with HPV awareness [12]. In Saudi Arabia, several studies targeting various populations in different regions reported unsatisfactory knowledge about HPV, pap smears, and the HPV vaccine [13,14,15,16,17,18,19]. While most local studies targeted the female population and examined HPV awareness mainly in the context of cervical cancer [18,19], one study targeting male medical students alone revealed low HPV knowledge [15]. Another study targeting both males and females from different regions in Saudi Arabia reported poor HPV knowledge among both genders, but more so among males [14].

With the exception of the aforementioned studies, little is known about gender differences in HPV awareness and knowledge among Saudi university students, let alone students from health colleges. The aim of this study was to determine HPV awareness and knowledge among male and female students in health colleges at King Saud University (KSU), Riyadh, and to compare awareness and knowledge across sociodemographic characteristics.

## 2. Materials and Methods

### 2.1. Study Design and Setting

We performed a cross-sectional survey-based study at the five health colleges: College of Medicine, College of Dentistry, College of Pharmacy, College of Applied Medical Sciences, and College of Nursing at KSU, Riyadh, Saudi Arabia. The sample size for this study was calculated to be 377 participants, using Raosoft, Inc. (Seattle, WA, USA) (http://www.raosoft.com/samplesize.html) (accessed on 26 July 2022), with 95% confidence and an error rate of 5%. Nevertheless, we included more responses until we reached 403 participants to make the study more meaningful.

### 2.2. Data Collection

Data were collected over two months, from November to December 2022, using a self-administered 28-item questionnaire, adapted from a study that surveyed male medical students in Jeddah, Saudi Arabia [15], who share similar cultural and medical backgrounds as our target population. A pilot study with 23 participants was conducted to test the questions’ clarity and the time needed to answer them. However, the results of the pilot study were not included in the final analysis. The first section comprised questions on the following sociodemographic characteristics: age, gender, nationality, marital status, cumulative grade point average (GPA), smoking status, hepatitis B (HBV) vaccination, and history of STIs. To investigate the awareness of HPV, participants were asked whether or not they had heard of HPV. If their answer was yes, they were transferred to the second section which comprised 17 “True/False/I Don’t Know” questions about HPV knowledge, formulated and validated by Waller et al. [20]. Each correct response was given a score of 1, while each incorrect or “I Don’t Know” response was given a 0 score; therefore, the total maximum possible score was 17. Based on the percentage of scores achieved by the participants, the knowledge scores were grouped into the following: good (>75%), fair (50–75%), and poor (<50%) [21].

### 2.3. Procedure for Data Collection

After we received the ethical approval, the survey link, which was prepared using Google forms, was distributed to the participants. Eligibility criteria were being an undergraduate student and studying at a health college at KSU. Postgraduate students were excluded. Data collectors from all of these health colleges were recruited to distribute the link to their classmates and through social media.

### 2.4. Ethical Consideration

Participants were aware of the study’s purpose and were asked voluntarily to fill out the survey after informed consent. Responses were completely anonymous and confidential. The study was ethically approved by the Institutional Review Board, KSU (ref. No. 22/0843/IRB). All data were kept confidential and were available only to the research team.

### 2.5. Statistical Analysis

We calculated percentages for categorical variables, while mean and standard deviation (SD) were calculated for the total knowledge scores. To assess the association of awareness with sociodemographic characteristics, we conducted a logistic regression model using binary awareness (yes vs. no) as the dependent variable, while age group, gender, college, history of HBV vaccination, history of STIs, and smoking status were independent variables, for which we report adjusted odds ratios (AORs) and 95% confidence intervals (CIs). To assess the association of HPV knowledge with the covariates, we conducted linear regression analysis using the knowledge score as the dependent variable, and the other covariates as independent variables, for which we report adjusted beta estimates and 95% CIs. The independent variables were added to the models because of their predictive properties based on the previous literature [15,22,23,24]. The data were analyzed using the Statistical Package for Social Sciences (SPSS) version 26 (Armonk, NY, USA) for IBM.

## 3. Results

### 3.1. Sociodemographic Characteristics of the Respondents

The survey was distributed to 450 students, out of which 403 (89.6%) responded with a completion rate of 100%, of whom 52.6% were female and 47.4% were male. Most of the respondents were Saudi nationals (98.5%) and belonged to the age group of 21–23 years (54.5%). Out of all the participating health colleges, the highest responses came from students at the College of Applied Medical Sciences (42.2%). The majority of respondents (98%) were single. Overall, 50.9% of the students had previously received the HBV vaccine and 2.5% had a history of STI (Table 1).

### 3.2. HPV Awareness and Knowledge

#### 3.2.1. Awareness of HPV

**Figure 1:**
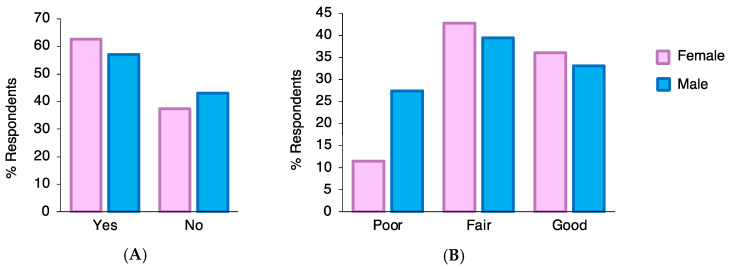
Awareness and knowledge of HPV among male and female students. Chi-square descriptive comparative test was applied. (**A**) Participants were asked whether they had previously heard of HPV. The number of participants responding with “Yes” or “No” responses are depicted as percentages (%). Males’ awareness of HPV was less than females’ (*p*-value = 0.003). (**B**) Participants answering yes to the question in (**A**) (N = 242) were asked a series of factual questions on HPV infection to assess the level of their knowledge about HPV. Knowledge scores were grouped into the following: good (>75%), fair (50–75%), and poor (<50%). The knowledge categories were not significantly different between male and female students (*p*-value = 0.502).

Out of the 403 respondents, 161 (40%) responded that they had never heard of HPV. Awareness of HPV was significantly lower among males (57.1%) compared to female students (62.7%) (*p*-value = 0.003) (Figure 1A). When comparing awareness across colleges, students from the College of Medicine had a significantly higher proportion of awareness (91.8%) compared to the rest of the colleges (X^2^ = 101.4; df = 4; *p*-value < 0.001). They were followed by the College of Dentistry (89.2%), Pharmacy (77.2%), Nursing (50.0%), and Applied Medical Sciences (35.3%). Because the total number of students reporting awareness of HPV was 242 (60%), only 242 responded to the section regarding knowledge about HPV.

#### 3.2.2. Knowledge about HPV

Out of the 242 students responding to knowledge questions, the frequency of female students exhibiting fair or good knowledge about HPV was greater than that among males, yet not statistically significant (*p*-value = 0.502) (Figure 1B). The overall mean knowledge score among these students was 8.8 (SD = 3.6). There was no significant difference between the mean knowledge scores for males (8.7, SD = 3.9) and females (8.9, SD = 3.5) (*p*-value = 0.675). With respect to individual knowledge questions, the highest proportion of correct responses was recorded for both genders in the item “HPV can be passed on during sexual intercourse”, with 77.1% of males and 83.5% of females answering it correctly. On the other hand, the lowest proportion of correct responses for both genders was recorded in the item “HPV usually doesn’t need any treatment”, with only 11.9% of males and 13.5% of females responding correctly (Table 2). It is worth mentioning that around 50% of the students from both genders believed that HPV infection can be treated with antibiotics, and around 60% thought that HPV can cause HIV.

#### 3.2.3. Association of Awareness and Knowledge with Sociodemographic Characteristics

Results from binary analyses suggest that the respondents’ level of knowledge regarding HPV was significantly associated with their age (χ^2^ = 40.293, *p* < 0.001) and college type (χ^2^ = 34.582, *p* < 0.001). Interestingly, there was a higher level of knowledge about HPV among students who had previously taken the HBV vaccine (χ^2^ = 11.926, *p* < 0.001) (Table 3).

As shown in Table 3, the majority of students were Saudi and single. Thus, these two variables were not included in regression analyses. Compared to students who were between 18 and 20 years, the odds for being aware of HPV were around 3-fold among students aged 21 to 23 years (AOR = 2.99; 95% CI = 1.71, 5.22), and were around 4-fold for students between the ages of 24 and 26 years (AOR = 4.13; 95% CI = 1.03, 16.54), suggesting that awareness about HPV may be associated with increasing age. However, this trend was not observed with the age group of 27 years or older (Table 4). Although the odds for awareness of HPV were lower among male students compared to female students, this point-estimate was not statistically significant. Students enrolled in medical college seemed to have greater odds of awareness compared to those from other colleges. This was particularly significant for students from the applied medical sciences, in which their odds of awareness were 89% lower than those for students in medical college (AOR = 0.11; 95% CI = 0.04, 0.29), and from the College of Nursing who had 90% lower odds (AOR = 0.10; 95% CI = 0.04, 0.29). Additionally, having an HBV vaccine seemed to significantly increase the odds of awareness (AOR = 2.10; 95% CI = 1.21, 3.64). Surprisingly, smoking was associated with around 5-fold odds of having good knowledge about HPV (AOR = 4.95; 95% CI = 1.84, 13.31). Finally, and although not statistically significant, having a history of STI was associated with lower odds of awareness about HPV.

Compared to students who were aged 18 to 20 years, on average, students aged 21 to 23 years had higher knowledge scores by 1.59 points (β = 1.59; 95% CI = 0.44, 2.74). On the other hand, and although not statistically significant, older students had lower knowledge scores (Table 5). As with awareness, the mean knowledge scores for students from the colleges of pharmacy, applied medical sciences, nursing, and dentistry were at least 2 points lower than that for students from the College of Medicine. However, this estimate was lower and not statistically significant for students from the College of Dentistry (β = −1.37; 95% CI = −2.79, 0.04). As with what was reported from bivariate analysis, on average, the knowledge score for males was not significantly lower than that for females when controlling for other covariates. Moreover, having the HBV vaccination and having an STI were not significantly associated with a change in knowledge score.

## 4. Discussion

Because HPV infection is a preventable disease, it is crucial to fill the gap in knowledge for future healthcare providers before their enrollment in the workforce. To do so, we evaluated the HPV knowledge in health students from the five health colleges at KSU in the central province of Saudi Arabia. The response rate was 89.6%, and the students who did not respond did not do so mainly because they were not students at any health colleges at KSU or did not agree to participate. Those who responded answered all the survey questions completely. Findings from the current study can be summarized in the following points. First, 40% of the students in our study reported not hearing about HPV. Additionally, the proportion of awareness was significantly greater among females compared to males. This trend was corroborated by the results from multivariate analyses, albeit not statistically significant. Second, the average HPV knowledge scores were only 8.9 and 8.7 for females and males, respectively. Third, we could not find significant differences in knowledge correctness or knowledge scores between male and female students. Fourth, compared to the youngest student group, students between the ages of 21 and 26 had greater odds of awareness of HPV, controlling for other student characteristics. Fifth, when controlling for other covariates, the odds of awareness were greater for medical students compared to students from all other health specialties, as were the mean knowledge scores. Finally, having the HBV vaccine seemed to increase the odds of awareness of HPV.

In this study, about 60% of the students were aware of HPV. Previous studies in Saudi Arabia reported awareness among college students ranging from 33.7% to 49.9% [25,26]. Even lower estimates were reported among university students in Morocco (10%) [27]. Although this estimate is greater than what has been reported in the Middle East and regionally, awareness among our study population still falls short compared to Western college students, in which awareness is reported as high as 95.3% [28]. Thus, HPV infection and vaccination awareness should be raised in local universities for both women and men.

Our study suggests that the overall awareness is greater among females. This comes in agreement with several international studies [27,28,29,30,31]. Possible explanations for this discrepancy could be that the complications of HPV infection are more commonly manifested in women, in addition to the recent regional introduction of the HPV vaccination into the vaccination schedule of adolescent girls, but not boys. Although awareness significantly differed between males and females on bivariate analysis, this significant association was not maintained through multivariate analysis. Additionally, we could not find significant differences between the genders in terms of knowledge scores. This is contrary to the worldwide belief that women consistently have better knowledge about HPV compared to men [32].

To the best of our knowledge, our study is the only local study to have compared HPV awareness and knowledge among university students of both genders within the central region of Saudi Arabia. A previous study conducted in the central province by AlShaikh et al. reported a lack of knowledge and misinformation regarding HPV among Saudi female university students alone, which corresponds with what we report [33]. Similar studies have also been published from the western [15], north-western [19], and south-western [34] regions of Saudi Arabia, reporting a lower prevalence of HPV awareness. For example, the study that was conducted among male medical students in Jeddah found that only 70% had ever heard of HPV [15]. Another study reported that 73% of medical students had heard of HPV [26].

Students in the College of Medicine had the highest proportion of awareness about HPV (91.8%) and scored the highest on HPV knowledge. Moreover, they had greater odds of awareness and knowledge about HPV compared to students from other health colleges. This has been repeatedly observed in other regional studies [15,26,29]. Perhaps the rigorous exposure of medical students to infectious diseases in their curriculum might explain this observation. Raising awareness of HPV is needed in all health colleges.

Awareness of HPV was more likely among students of greater age groups compared to those who were between 18 and 20. This has also been reported in a study from Turkey [35]. However, this trend was not observed when comparing knowledge scores across the age groups. This contradicts the notion that knowledge about HPV increases with age among university students [36]. On the other hand, one study that sampled the general population reported a slight decrease in knowledge with each one-year increase in age [37]. In both studies, age was measured as a continuous variable, unlike our categorical recording of age in the questionnaire, which may have contributed to this discrepancy.

We observed that having received the HBV vaccine increased the odds of HPV awareness and knowledge. This observation corresponds with previous reports that used the same methodology [15]. Not only does HBV vaccination increase ones’ awareness and knowledge of HPV, but it has been associated with a reduction in HPV vaccination hesitancy [15]. Moreover, HBV vaccination may be an indicator for preventative care, which increases the chance of awareness. Targeting both viruses with vaccination programs may help increase community acceptance for vaccine reception, aiding in the simultaneous prevention and control of both HBV and HPV infections.

Because the subject of STIs is greatly affected by society, in a culturally and religiously conservative country such as Saudi Arabia, sexual education is not well-provided during school years. Thus, we find a need to fill the gap in knowledge in the community, starting by improving the overall awareness and knowledge of future healthcare providers. This is crucial for preparing health students for their future role as public health educators and potential immunizers.

The current study has highlighted the urgent need to increase HPV awareness and knowledge among health college students in Saudi Arabia. This could be achieved by implementing explicit HPV educational modules in their undergraduate programs. Our study is not free of limitations. For example, we only sampled from one university in the central province of Saudi Arabia. Therefore, our findings cannot be generalized to the entire Saudi university population, and larger nationwide studies may be needed. Secondly, only a small proportion of students reported having a history of STIs (10), which could reflect underreporting for this estimate due to social desirability. Because participation in this study was voluntary, students who showed interest in the study may be more health aware in general. This could have potentially introduced selection bias that may have overestimated our outcomes. Furthermore, our model only controlled for the measured variables, so we cannot exclude confounding by unmeasured variables that are important in assessing the association between gender and HPV awareness and knowledge. Thus, the reader should interpret the results with these points of caution. Despite these limitations, our study sheds light on the understudied level of awareness and knowledge about HPV among health college students in the heavily conservative Saudi society. Health college students play a crucial role in spreading awareness about HPV infection and other STIs in their communities. Improving their knowledge about such diseases and empowering their community health-educational skills is fundamental to STI control and prevention.

## 5. Conclusions

The current study suggests a 40% gap in awareness of HPV among university students and more so among males. Additionally, students in health disciplines exhibited lower levels of awareness and knowledge compared to medical students. The data presented in this study provide a point of reference around the level of HPV knowledge among KSU health college students, which can be used to frame an effective awareness program, especially among male students. We recommend further incorporation of HPV infection, its complication, and vaccine benefits into the curricula of all health college university students. Applying university-wide interventions and educational campaigns can boost HPV knowledge and awareness, which can ultimately promote community adoption of HPV vaccination.

## Figures and Tables

**Table 1:** Sociodemographic characteristics of the respondents.

Item	Total (N = 403)		Male (N = 191)		Female (N = 212)	
	N	%	N	%	N	%
Age						
18–20 years	174	41.4	79	44.8	95	43.2
21–23 years	204	54.5	104	47.2	100	50.6
24–26 years	22	3.7	7	7.1	15	5.5
27 or more	3	0.5	1	0.9	2	0.7
In which college are you studying?						
Medicine	85	21.1	41	21.5	44	20.8
Pharmacy	57	14.1	50	26.2	7	3.3
College of Applied Medical Sciences	170	42.2	83	43.5	87	41.0
Nursing	54	13.4	6	3.1	48	22.6
Dentistry	37	9.2	11	5.8	26	12.3
Nationality						
Saudi	397	98.5	188	98.4	209	98.6
Non-Saudi	6	1.5	3	1.6	3	1.4
Marital Status						
Single	395	98.0	188	98.4	207	97.6
Married	6	1.5	3	1.6	3	1.4
Divorced	2	0.9			2	.9
Cumulative grade point average (GPA)						
4 or more	290	64.4	118	61.8	172	81.1
Less than 4	114	25.1	73	38.2	40	18.9
Do you smoke?						
Yes	55	13.6	42	22.0	13	6.1
No	348	86.4	149	78.0	199	93.9
Did you take Hepatitis B Vaccine?						
Yes	205	50.9	84	44.0	121	57.1
No	198	49.1	107	56.0	91	42.9
Do you have a history of any sexually transmitted infections?						
Yes	10	2.5	4	2.1	6	2.8
No	393	97.5	187	97.9	206	97.2
Have you heard of the human papillomavirus (HPV)?						
Yes	242	60.0	109	57.1	133	62.7
No	161	40.0	82	42.9	79	37.3

Note: the number of responses is denoted as N with the corresponding percentages (%).

**Table 2:** Distribution of the respondents regarding knowledge.

Item	Male (N = 109)				Female (N = 133)				*p*-Value
	Incorrect/ Did Not Know		Correct		Incorrect/ Did Not Know		Correct		
	N	%	N	%	N	%	N	%	
Human papillomavirus (HPV) is very rare	46	42.2	63	57.8	43	32.3	90	67.7	0.140
HPV always has visible signs or symptoms	57	52.3	52	47.7	63	47.4	70	52.6	0.518
HPV can cause cervical cancer in females	26	23.6	83	76.1	23	17.3	110	82.7	0.260
HPV can cause oropharyngeal cancers in males	62	56.9	47	43.1	88	66.2	45	33.8	0.146
HPV can be passed on by genital skin-to-skin contact	46	42.2	63	57.8	49	36.8	84	63.2	0.429
There are many types of HPV	73	67.0	36	33.0	89	66.9	44	30.1	0.435
HPV can cause HIV/AIDS	67	61.5	42	38.5	89	66.9	44	33.1	0.419
HPV can be passed on during sexual intercourse	25	22.9	84	77.1	22	16.5	111	83.5	0.253
HPV can cause genital warts	36	33.0	73	67.0	42	31.6	91	68.4	0.890
Men cannot get HPV	28	25.7	81	74.3	38	28.6	95	71.4	0.665
Using condoms reduces the risk of getting HPV	38	34.9	71	65.1	41	30.8	92	69.2	0.582
HPV can be cured with antibiotics	55	50.5	54	49.5	60	45.1	73	54.9	0.439
Having many sexual partners increases the risk of getting HPV	25	22.9	84	77.1	19	14.3	114	85.7	0.095
HPV usually doesn’t need any treatment	96	88.1	13	11.9	115	86.5	18	13.5	0.847
Most sexually active people will get HPV at some point in their lives	77	70.6	32	29.4	94	70.7	39	29.3	0.553
A person could have HPV for many years without knowing it	42	38.5	67	61.5	41	30.8	92	69.2	0.223
Having sexual intercourse at an early age increases the risk of getting HPV	52	47.7	57	52.3	69	51.9	64	48.1	0.605

Note: the number of responses is denoted as N with the corresponding percentages (%).

**Table 3:** Relationship between the sociodemographic characteristics and the HPV knowledge levels.

Sociodemographic Characteristics	Knowledge about HPV						
	Poor (<50%)		Fair (50–75%)		Good (>75%)		*p*-Value
	N	%	N	%	N	%	
Age							
18–20 years	31	53.4	20	20.0	8	9.5	<0.001
21–23 years	23	39.7	73	73.0	67	79.8	
24–26 years	4	6.9	6	6.0	9	10.7	
27 or more	0	0.0	1	1.0	0	0.0	
In which college are you studying?							
Medicine	8	13.8	29	29.0	41	48.8	<0.001
Pharmacy	14	24.1	16	16.0	14	16.7	
College of Applied Medical Sciences	22	37.9	22	22.0	16	19.0	
Nursing	11	19.0	13	13.0	3	3.6	
Dentistry	3	5.2	20	20.0	10	11.9	
Gender							
Male	30	51.7	43	43.0	36	42.9	0.502
Female	28	48.3	57	57.0	48	57.1	
Nationality							
Saudi	47	98.3	98	98.0	83	98.8	0.911
Non-Saudi	1	1.7	2	2.0	1	1.2	
Marital Status							
Single	57	98.3	99	99.0	83	98.8	
Married	1	1.7	1	1.0	1	1.2	0.923
Divorced	0	0.0	0	0.0	0	0.0	
Cumulative grade point average (GPA)							
4 or more	143	60.3	121	98.0	26	86.7	<0.001
Less than 4	94	39.7	15	11.0	4	13.3	
Do you smoke?							
Yes	6	10.3	12	12.0	16	19.0	0.254
No	52	89.7	88	88.0	68	81.0	
Did you take hepatitis B Vaccine?							
Yes	28	48.3	70	70.0	63	75.0	<0.001
No	30	51.7	30	30.0	21	25.0	
Do you have a history of any sexually transmitted infections?							
Yes	0	0.0	2	2.0	2	2.4	0.516
No	58	100.0	98	98.0	82	97.6	

Note: the number of responses denoted as N, with the corresponding percentages (%).

**Table 4:** Prediction of HPV awareness by sociodemographic characteristics.

Sociodemographic Characteristics	Awareness of HPV	
	AOR	95% CI
Age		
18–20 years	Ref	
21–23 years	2.99	1.71, 5.22
24–26 years	4.13	1.03, 16.54
27 or more	0.47	0.03, 7.31
In which college are you studying?		
Medicine	Ref	
Pharmacy	0.42	0.14, 1.24
College of Applied Medical Sciences	0.11	0.04, 0.29
Nursing	0.10	0.04, 0.29
Dentistry	0.48	0.12, 1.91
Gender		
Male	0.59	0.32, 1.08
Female	Ref	
Cumulative grade point average (GPA)		
4 or more	1.54	0.86, 2.77
Less than 4	Ref	
Do you smoke?		
Yes	1.19	0.55, 2.58
No	Ref	
Did you take hepatitis B Vaccine?		
Yes	2.10	1.21, 3.64
No	Ref	
Do you have a history of any sexually transmitted infections?		
Yes	0.41	0.08, 2.01
No	Ref	

Notes: AOR = adjusted odds ratio. CI = confidence interval.

**Table 5:** Prediction of HPV knowledge score by sociodemographic characteristics.

Sociodemographic Characteristics	HPV Knowledge Score	
	Beta Estimate	95% CI
Age		
18–20 years	Ref	
21–23 years	1.59	0.44, 2.74
24–26 years	−0.13	−2.02, 1.76
27 or more	−1.02	−7.94, 5.90
In which college are you studying?		
Medicine	Ref	
Pharmacy	−2.18	−3.52, −0.85
College of Applied Medical Sciences	−2.22	−3.62, −0.82
Nursing	−2.84	−4.43, −1.25
Dentistry	−1.37	−2.79, 0.04
Gender		
Male	−0.91	−1.96, 0.14
Female	Ref	
Cumulative grade point average (GPA)		
4 or more	0.34	−0.97, 1.65
Less than 4	Ref	
Do you smoke?		
Yes	1.72	0.32, 3.13
No	Ref	
Did you take hepatitis B Vaccine?		
Yes	0.51	−0.51, 1.54
No	Ref	
Do you have a history of any sexually transmitted infections?		
Yes	1.66	−1.84, 5.16
No	Ref	

Notes: CI = confidence interval.

## Data Availability

The data that support the findings of this study are available upon request from the corresponding author E.A.
